# Improved navigator-gated motion compensation in cardiac MR using additional constraint of magnitude of motion-corrupted data

**DOI:** 10.1186/1532-429X-14-S1-P245

**Published:** 2012-02-01

**Authors:** Jaime L Shaw, Mehdi H Moghari, Mehmet Akcakaya, Raymond H Chan, Warren J Manning, Reza Nezafat

**Affiliations:** 1 Medicine, Beth Israel Deaconess Medical Center and Harvard Medical School, Boston, MA, USA; 2 Radiology, Beth Israel Deaconess Medical Center and Harvard Medical School, Boston, MA, USA

## Background

In conventional prospective respiratory navigator (NAV) acquisitions, 40-60% of the acquired data are discarded resulting in low efficiency and long scan times [1,2]. Compressed-sensing Motion Compensation (CosMo) has a shorter fixed scan time by acquiring the full inner k-space and estimating the NAV-rejected outer k-space lines [3]. Respiratory motion will mainly manifest itself as phase variation in the acquired k-space data. We sought to determine if the addition of the magnitude of the rejected k-space lines as a constraint in image reconstruction will improve the performance of CosMo.

## Methods

To investigate the variability of the magnitude of k-space lines at different respiratory phases, free-breathing, ECG-triggered, targeted right coronary images with multiple averages were acquired from 10 healthy adult subjects. Magnitude variability was investigated quantitatively by calculating the cross-correlation between accepted and rejected k-space lines.

CosMo was implemented retrospectively on one acquisition from each subject. The inner k-space (31 ky by 7 kz lines) was filled with lines acquired within the 5 mm gating window from all acquisitions. The outer k-space was then filled only with lines from the first average acquired within the 5 mm gating window, resulting in an undersampled k-space with a fully sampled center. For reliable image reconstruction with CosMo, 10-20% of the inner k-space must be fully-sampled. The missing outer k-space lines were then estimated using LOST with an additional magnitude constraint within each estimation iteration or in the final iteration for each coil [4]. The results were compared with prospective NAV-gating with a gating window of 5 mm and CosMo reconstruction without the magnitude constraint.

## Results

**Figure 1:**
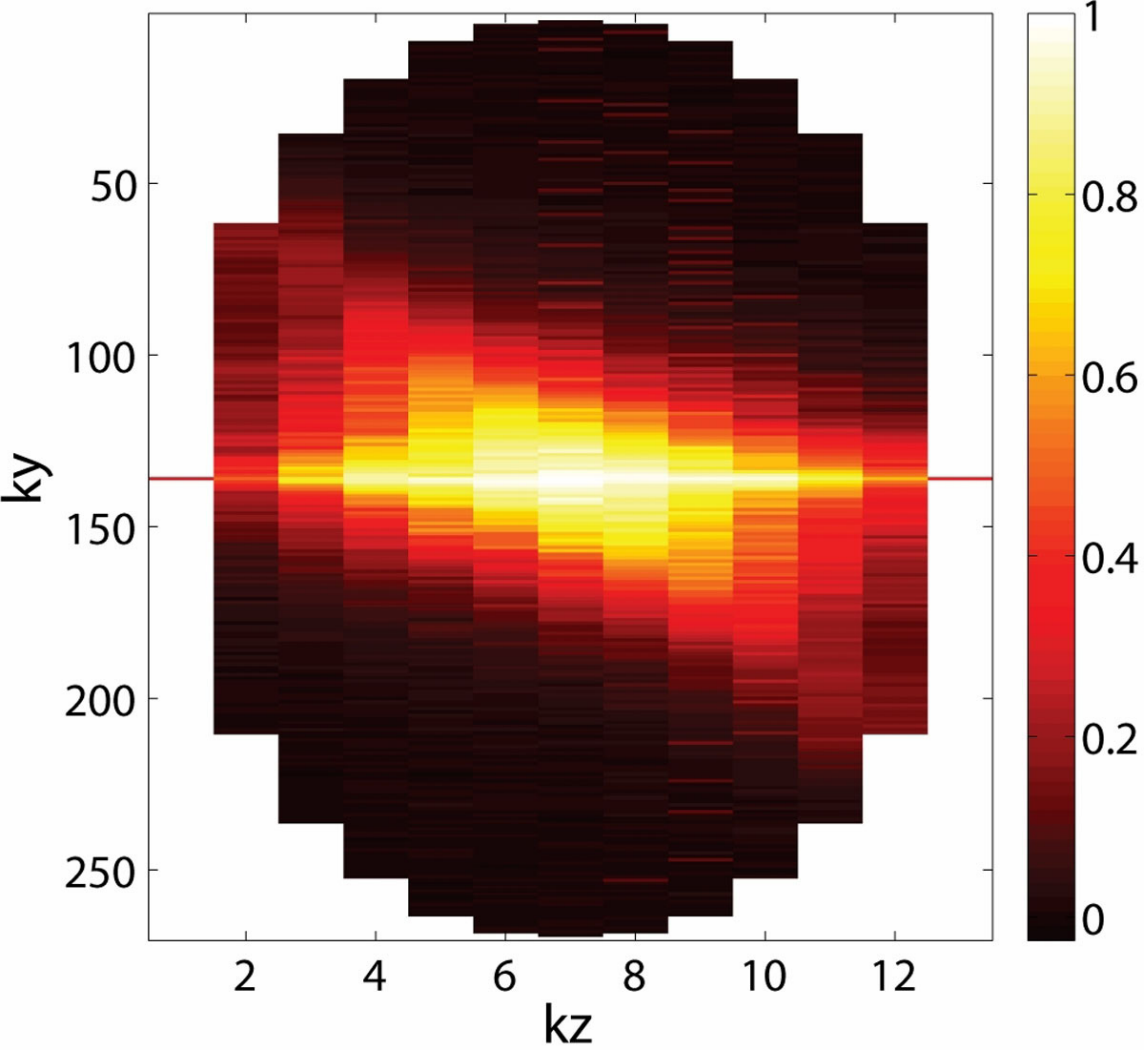
Cross-correlation between the accepted and rejected k-space lines averaged over all coils for each ky-kz position.

**Figure 2:**
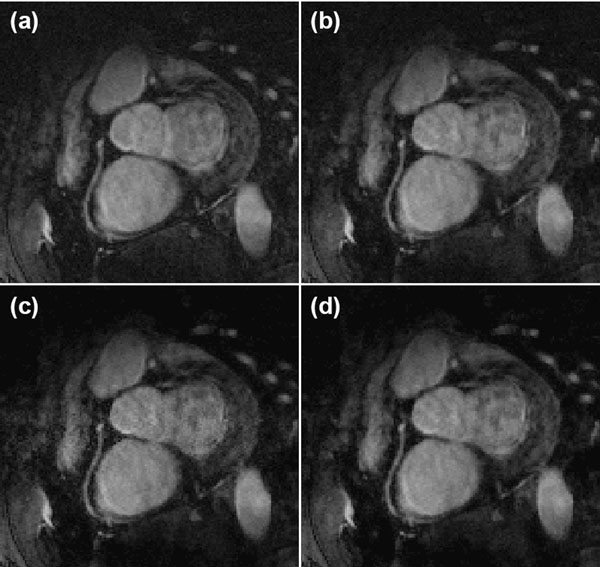
RCA images from a healthy subject reconstructed using 5-mm navigator gating (a), CosMo reconstruction without the magnitude constraint (b), CosMo reconstruction with the magnitude constraint added to each estimation iteration (c), and CosMo reconstruction with the magnitude constraint added in the final iteration (d).

Figure 1 shows the cross-correlation between the accepted and worst rejected k-space lines for each position. The correlation is close to 1 at the center of k-space where the majority of image information is contained, indicating low variability in magnitude information at different respiratory phases. Figure 2 shows right coronary images acquired using a) fully-sampled, 5-mm gated data, b) the original CosMo, and CosMo with the additional magnitude constraint c) inside each iteration and d) in the final iteration. The relative signal-to-noise in the left ventricle blood pool is: 30.71±6.5; 40.32±14.2; 53.9±26.8; 56.8±25.9 for each reconstruction, respectively. Significant differences (p<0.05) are present for all measurements except between the original CosMo and the CosMo image with the magnitude constraint in each iteration (p=0.09).

## Conclusions

The addition of the magnitude of rejected lines, readily available in all navigator-gated scans, as a constraint in CosMo results in improved image quality as measured by relative SNR.

## Funding

NIH R01EB008743-01A2.
